# Single-molecule study reveals Hmo1, not Hho1, promotes chromatin assembly in budding yeast

**DOI:** 10.1128/mbio.00993-23

**Published:** 2023-07-11

**Authors:** Mengxue Wang, Jinghua Li, Yong Wang, Hang Fu, Haoning Qiu, Yanying Li, Ming Li, Ying Lu, Yu Vincent Fu

**Affiliations:** 1 State Key Laboratory of Microbial Resources, Institute of Microbiology, Chinese Academy of Sciences, Beijing, China; 2 College of Life Sciences, University of Chinese Academy of Sciences, Beijing, China; 3 Beijing National Laboratory for Condensed Matter Physics, Institute of Physics, Chinese Academy of Sciences, Beijing, China; 4 Wenzhou Institute, University of Chinese Academy of Sciences, Wenzhou, Zhejiang, China; 5 School of Physics, University of Chinese Academy of Sciences, Beijing, China; 6 Songshan Lake Materials Laboratory, Dongguan, Guangdong, China; 7 Savaid Medical School, University of Chinese Academy of Sciences, Beijing, China; Harvard Medical School, Boston, Massachusetts, USA

**Keywords:** chromatin assembly, linker histone H1, Hmo1, total internal reflection fluorescence microscopy, magnetic tweezers

## Abstract

Linker histone H1 plays a crucial role in various biological processes, including nucleosome stabilization, high-order chromatin structure organization, gene expression, and epigenetic regulation in eukaryotic cells. Unlike higher eukaryotes, little about the linker histone in *Saccharomyces cerevisiae* is known. Hho1 and Hmo1 are two long-standing controversial histone H1 candidates in budding yeast. In this study, we directly observed at the single-molecule level that Hmo1, but not Hho1, is involved in chromatin assembly in the yeast nucleoplasmic extracts (YNPE), which can replicate the physiological condition of the yeast nucleus. The presence of Hmo1 facilitates the assembly of nucleosomes on DNA in YNPE, as revealed by single-molecule force spectroscopy. Further single-molecule analysis showed that the lysine-rich *C*-terminal domain (CTD) of Hmo1 is essential for the function of chromatin compaction, while the second globular domain at the *C*-terminus of Hho1 impairs its ability. In addition, Hmo1, but not Hho1, forms condensates with double-stranded DNA via reversible phase separation. The phosphorylation fluctuation of Hmo1 coincides with metazoan H1 during the cell cycle. Our data suggest that Hmo1, but not Hho1, possesses some functionality similar to that of linker histone in *Saccharomyces cerevisiae*, even though some properties of Hmo1 differ from those of a canonical linker histone H1. Our study provides clues for the linker histone H1 in budding yeast and provides insights into the evolution and diversity of histone H1 across eukaryotes.

**IMPORTANCE:**

There has been a long-standing debate regarding the identity of linker histone H1 in budding yeast. To address this issue, we utilized YNPE, which accurately replicate the physiological conditions in yeast nuclei, in combination with total internal reflection fluorescence microscopy and magnetic tweezers. Our findings demonstrated that Hmo1, rather than Hho1, is responsible for chromatin assembly in budding yeast. Additionally, we found that Hmo1 shares certain characteristics with histone H1, including phase separation and phosphorylation fluctuations throughout the cell cycle. Furthermore, we discovered that the lysine-rich domain of Hho1 is buried by its second globular domain at the *C*-terminus, resulting in the loss of function that is similar to histone H1. Our study provides compelling evidence to suggest that Hmo1 shares linker histone H1 function in budding yeast and contributes to our understanding of the evolution of linker histone H1 across eukaryotes.

## INTRODUCTION

In eukaryotes, the basic unit of chromatin is the nucleosome, which is tightly connected by linker DNA (1 - 3). The linker histone H1 preferentially binds at the linker DNA entry/exit site and plays a critical role in nucleosome stability and chromatin compaction (4 - 8). Meanwhile, new functions of linker histone H1 have been discovered, such as epigenetic regulation, DNA replication, DNA repair, and genome stability (5, 7, 9 - 11). Compared to the core histones highly conserved among different species, histone H1 is more heterogeneous across species with a range of variants (12 - 14). In addition, H1 can be modified by multiple post-translational modifications, thereby performing precise functions in various biological processes (15). The tripartite structure of H1 consists of a short *N*-terminus, a conserved central globular domain, and a long intrinsically disordered lysine-rich *C*-terminus (16, 17). It has been reported that the *C*-terminal domain of H1 is necessary for regulating higher-order chromatin organization, possibly by modulating the intrinsic phase-separation property of chromatin (18 - 20).

Although the metazoan linker histone H1 has been extensively studied, many details of H1 in eukaryotic microorganisms remain unclear. The question of who functions as linker histone H1 in *Saccharomyces cerevisiae* has long been debated (21 - 24). Due to the highly homologous amino acid sequence to the globular domain of metazoan H1, Hho1 was characterized as histone H1 in budding yeast (25, 26). Nonetheless, many characteristics of Hho1 differ from those of histone H1 in metazoans. For instance, Hho1 contains two globular domains rather than one in metazoan H1 (27, 28). The stoichiometric ratio of Hho1 is calculated to be 0.027 molecules per nucleosome, much lower than 0.8 molecules per nucleosome in metazoans (29, 30). Hho1 negatively regulates the transcriptionally silent chromatin, whereas metazoan H1 often promotes heterochromatin formation (31 - 33). Notably, in the *hho1Δ* mutant, no significant alterations in yeast in cell morphology, chromatin structure, and DNA sensitivity to nuclease were observed (22, 25, 34).

Due to the suspicion that Hho1 is not the linker histone H1 in *Saccharomyces cerevisiae*, Hmo1, a member of the high mobility group B (HMGB) family, has been proposed to function as a linker histone (22, 23). The high mobility group (HMG) family is composed of three subfamilies, HMGA, HMGB, and HMGN (35). Recent studies have demonstrated that HMGB proteins can bind to the nucleosome entry/exit site, causing changes in chromatin structure and promoting transcription, achieved through the interaction of the acidic *C*-terminus of HMGB proteins with core histones (36 - 38).

Hmo1 contains two HMG domains followed by one lysine-rich *C*-terminal tail. Deleting the *HMO1* gene is not lethal in *S. cerevisiae* but results in growth defects, plasmid loss, as well as chromatin hypersensitivity to micrococcal nuclease (39). Distinct from the acidic *C*-terminal domain (CTD) present in many HMGB family proteins, the lysine-rich tail of Hmo1 is similar to the *C*-terminus of metazoan H1. Studies have shown that Hmo1 can bend and bridge DNA through the lysine-rich CTD. The CTD is also essential for protecting chromatin from digestion by micrococcal nuclease (22, 40, 41). Furthermore, Hmo1 is calculated to be approximately 19,000–25,000 molecules per cell, equivalent to 0.4 Hmo1 per nucleosome (42). Hmo1 is identified as a transcription factor involved in the transcription of *rDNA* genes with polymerase I (43, 44) and in the formation of transcription initiation protein complexes during polymerase II-related transcription (45, 46). Recent studies suggested that Hmo1 not only participates in transcriptional regulation but also in the formation and sustenance of chromatin structure (22, 47, 48). In addition, genome-level studies found that Hmo1 binds to the entire genome DNA by different occupancy, not just *rDNA* loci or genes encoding ribosomal proteins (44). However, these studies presented conflicting conclusions about the function of Hmo1. So far, most data about Hmo1 have been obtained only based on genetic studies, while direct biochemical evidence is still lacking.

Chromatin assembly is a highly dynamic process that involves histones, chaperones, and other regulating proteins (49 - 51). *In vitro* experiments with a single or several purified protein(s) might not be able to fully replicate the complex process of chromatin assembly under physiological conditions *in vivo*. To overcome this limitation, *Xenopus* egg extracts and *Drosophila* embryo extracts have been employed to study the physiological chromatin assembly (52 - 55). Similarly, we have developed yeast nucleoplasmic extracts (YNPE) as a competent biochemical system for chromatin assembly under physiological conditions *in vitro* (56, 57).

In this study, we utilized the YNPE to simulate the physiological conditions in yeast nuclei, combined with single-molecule approaches, to explore the functions of Hho1 and Hmo1 in chromatin assembly. We demonstrated that Hmo1, but not Hho1, involves in chromatin assembly in YNPE. The lysine-rich tail of Hmo1 is essential for its function in chromatin assembly. Moreover, we showed that Hmo1 is similar to metazoan histone H1 in terms of phase-separation property and phosphorylation during the cell cycle.

## RESULTS

### Hmo1, not Hho1, affects chromatin assembly in YNPE

**Fig 1:**
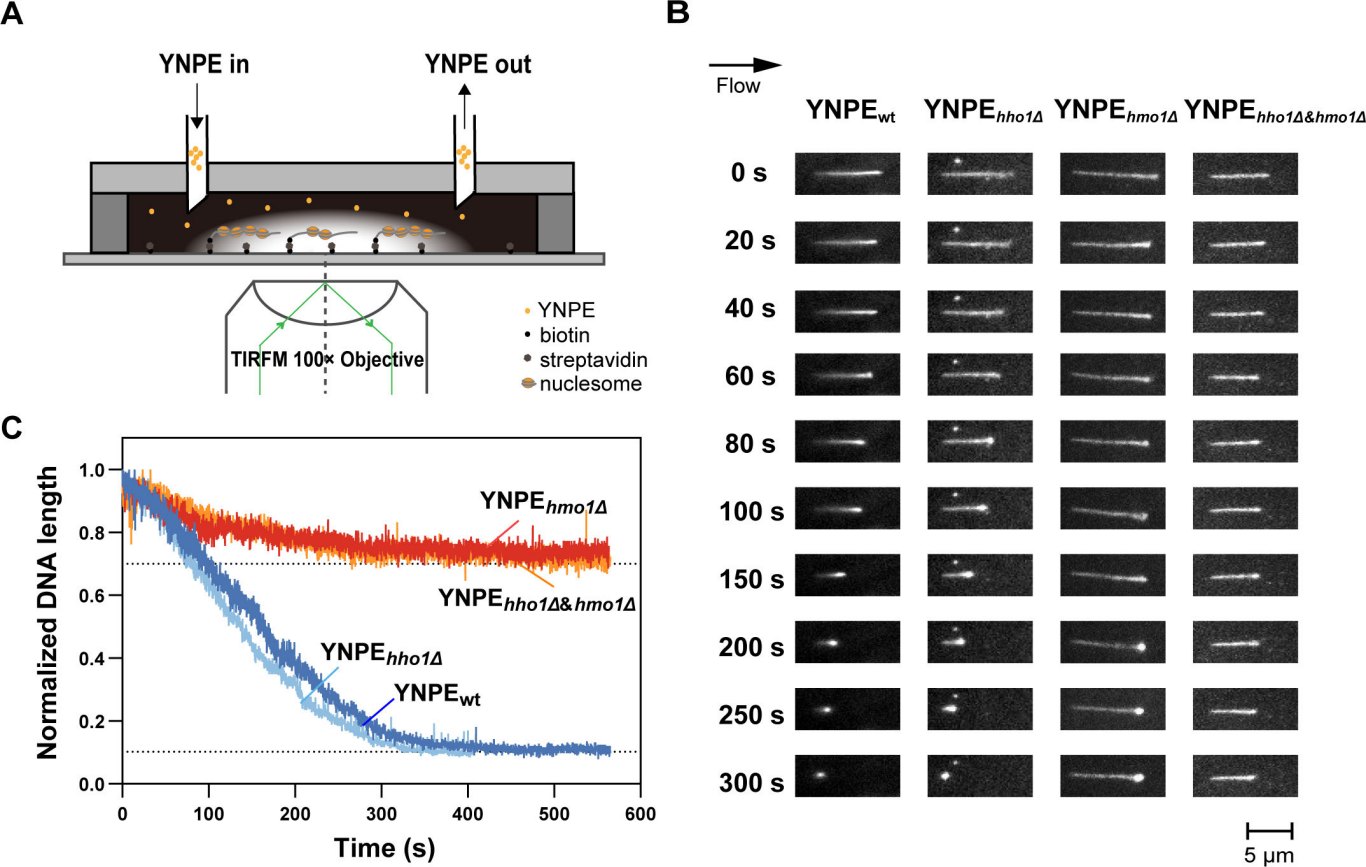
The role of Hmo1 and Hho1 in chromatin assembly. (**A**) Experimental setup using single-molecule total internal reflection fluorescence microscopy (TIRFM). λDNA molecules were immobilized on the surface of the flow cell via streptavidin-biotin interactions. Yeast nucleoplasmic extracts (YNPE), containing all soluble proteins in the yeast nuclei, were diluted with blocking buffer (20 mM Tris-HCl pH 7.5, 50 mM NaCl, 2 mM EDTA, 0.2 mg/mL BSA) and injected into the flow cell at 50 µL/min to assembly chromatin at room temperature. (**B**) The DNA compaction induced by YNPE_wt_, YNPE*_hho1Δ_*, YNPE*_hmo1Δ_*, and YNPE*_hho1Δ&hmo1Δ_*. Snapshots represent representative individual DNA molecules at specified time points in the time-lapse movie recorded for each type of YNPE. (**C**) Kinetic curves of λDNA compaction in YNPE_wt_ (dark blue), YNPE*_hho1Δ_* (blue), YNPE*_hmo1Δ_* (red), and YNPE*_hho1Δ&hmo1Δ_* (orange). The curves are plotted based on the average length of more than 30 individual DNA molecules measured in experiments.

YNPE, which contains all soluble proteins in the yeast nucleus, has been reported to support nucleosome formation and chromatin assembly under a simulated physiological condition (56, 57). One end of fluorescently stained λDNA was tethered on the surface of the flow cell via biotin-streptavidin interaction. With the Poiseuille flow of blocking buffer at a constant rate of 50 µL/min, the average extended length of the λDNA molecules was 11.86 ± 2.22 µm (*n* = 300), which was about 72% of the theoretical λDNA length (~16.49 µm) (Fig. 1A and B top panel; Fig. S1A). After the original YNPE (protein concentration was around 4.93 mg/mL) extracted from the wild-type yeast strains was introduced at a rate of 50 µL/min, DNA molecules retracted sharply and eventually shrunk into quasi-spherical dots in less than 10 seconds, which represented the biological process of chromatin assembly as described before (52, 53, 56). To ensure that the compression rate of the λDNA was within a suitable range for easy observation and improved the signal-to-noise ratio, we tested 1:200 and 1:2,000 dilutions of wild-type YNPE (protein concentration of 25 µg/mL and 2.5 µg/mL) (Fig. S1B). Using the 1:2,000 diluted wild-type YNPE (protein concentration of 2.5 µg/mL), the λDNA molecule retracted from the initial length to a dot (<1.5 µm in diameter) in around 400 seconds (Fig. 1B and C; Fig. S1B). In subsequent single-molecule experiments, all YNPEs were adjusted to the protein concentration of 2.5 µg/mL.

To investigate the effects of Hmo1 and Hho1 on chromatin assembly, we constructed *hmo1Δ* and *hho1Δ* strains, respectively. The DNA compaction behavior was monitored in YNPE prepared from these two strains. As shown in Fig. 1B and C, after immersion in the Hho1-deficient YNPE, λDNA shrunk quickly and eventually compacted into a dot, the same as what happened in the wild-type YNPE. The packing ratio (the initial DNA length divided by the packaged DNA length) was over 10. On the contrary, in the Hmo1-deficient YNPE, λDNA was incompletely compressed, only retracting to 70% of its original length with a packing ratio of less than 1.5, similar to what happened in the H1.8 depletion in the *Xenopus* egg extracts (55). Likewise, we observed the DNA compaction process in Hho1&Hmo1-deficient YNPE. DNA was packed in the Hho1&Hmo1-deficient YNPE with similar kinetics to the Hmo1-deficient YNPE (Fig. 1B and C). The results strongly suggested that Hmo1, but not Hho1, is essential for the chromatin assembly in YNPE.

### Hmo1 directly participates in chromatin assembly in YNPE

**Fig 2:**
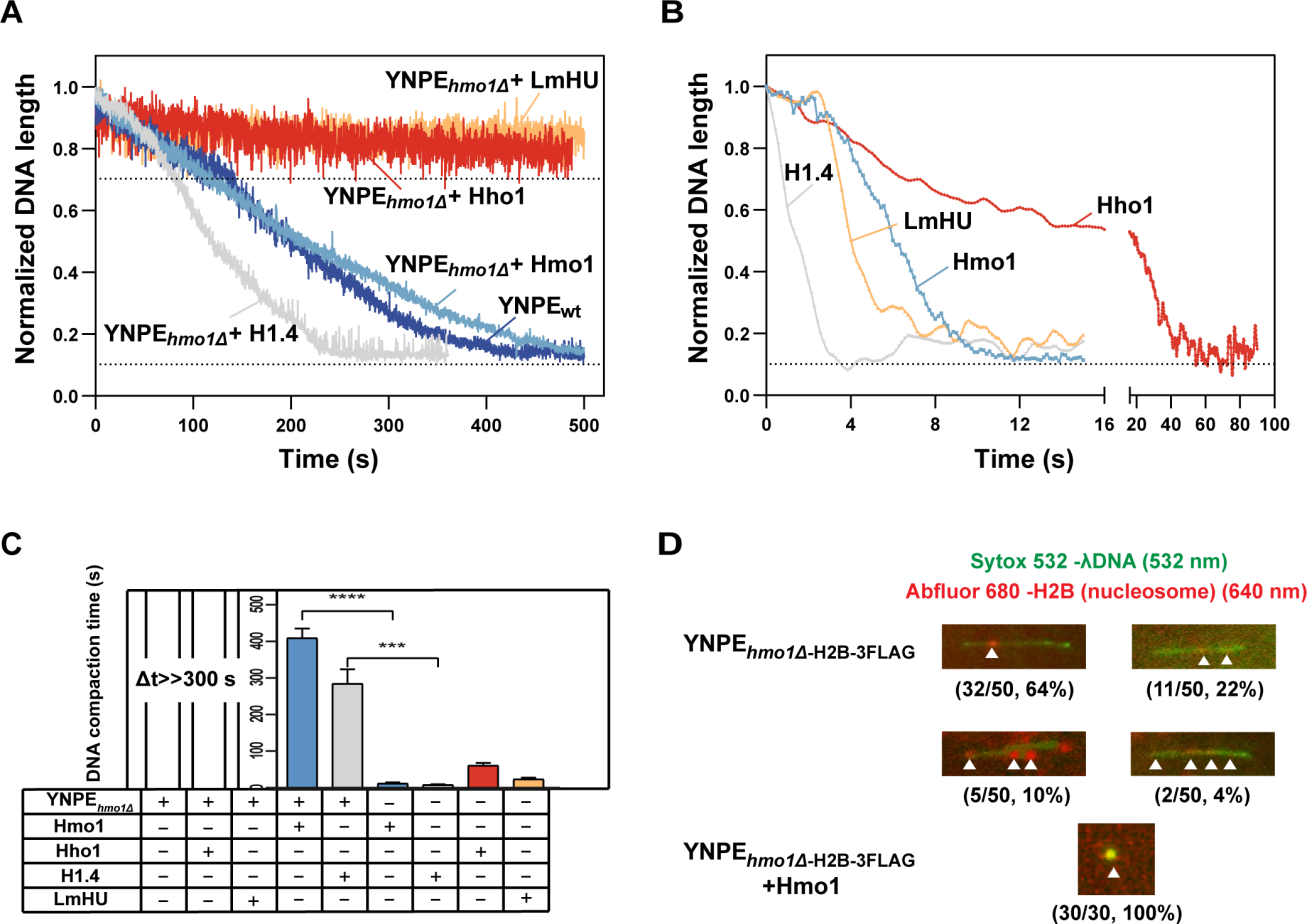
Hmo1 acts directly on chromatin assembly and facilitates further compaction of nucleosomes. (**A**) Kinetic analysis of supplementing four different purified proteins into YNPE*_hmo1Δ_* [YNPE*_hmo1Δ_* + H1.4 (gray), YNPE*_hmo1Δ_* + Hmo1 (light blue), YNPE*_hmo1Δ_* + Hho1 (red), YNPE*_hmo1Δ_* + LmHU (orange)]. The wild-type YNPE curve (dark blue) was used as a control. More than 20 DNA molecules were measured in each experiment. (**B**) Kinetic analysis of four different purified proteins without YNPE [H1.4 (gray), LmHU (orange), Hmo1 (blue), and Hho1 (red)]. More than 20 DNA molecules were measured in each experiment. (**C**) DNA compaction time under various conditions (*n* > 3, **** and *** means *P* value < 0.0001 and *P* value < 0.001, respectively). (**D**) Top: fluorescence imaging of nucleosomes on λDNA molecules in YNPE*_hmo1Δ-_*_H2B-3FLAG_. A total of 50 DNA molecules were counted, and the percentage of each representative image is shown in the parentheses. Bottom: fluorescence imaging of nucleosomes on λDNA molecule in YNPE*_hmo1Δ-_*_H2B-3FLAG_+ Hmo1. A total of 30 DNA molecules were counted, and the percentage of the representative image is shown in the parentheses.

Hmo1 has been identified as a transcription factor in *S. cerevisiae* (58). Knocking out *HMO1* affects the expression of a range of genes. Therefore, it remained the possibility that the effect of Hmo1 on chromatin assembly in YNPE was due to its regulatory effect on gene expression rather than direct involvement in chromatin assembly. To rule out this possibility, the purified protein Hmo1 was supplemented into the Hmo1-deficient YNPE. When purified Hmo1 was added to the YNPE*_hmo1Δ_* at a final concentration of 8.2 nM, the λDNA molecule resumed further retraction and finally contracted into a dot with kinetics similar to the wild-type YNPE [Fig. 2A, YNPE*_hmo1Δ_* + Hmo1 (light blue curve), YNPE_wt_ (dark blue curve)]. Moreover, the DNA retraction rate increased with the increasing concentration of Hmo1 (Fig. S2). When introducing 8.2 nM human linker histone H1.4 to the YNPE*_hmo1Δ_*, the compaction of λDNA was recovered in a faster kinetic manner compared to Hmo1 (Fig. 2A, gray curve). Given that both Hmo1 and H1.4 have been reported to have the ability to bend and bridge dsDNA *in vitro* (47, 59), we further investigated the effect of bacterial HU protein (derived from *Listeria monocytogenes*) on DNA compaction in YNPE*_hmo1Δ_*. Like Hmo1 and H1.4, HU can bridge and loop dsDNA *in vitro*, as demonstrated by the single-molecule magnetic tweezers experiments (Fig. S6). However, LmHU apparently does not partake in the chromatin assembly of the yeast cells. As shown by the orange curve in Fig. 2B, even adding 0.1 µM purified HU protein to YNPE*_hmo1Δ_* failed to induce further DNA compaction. This finding excluded the possibility that further DNA compaction in YNPE*_hmo1Δ_* was only due to the supplemented protein’s capacity to loop DNA or alter DNA elasticity. Likewise, supplementation with 8.2 nM purified protein Hho1 to YNPE*_hmo1Δ_* did not restore the DNA compression (Fig. 2A, red curve).

Furthermore, when the purified proteins Hmo1, Hho1, H1.4, and HU were introduced into the flow cell alone rather than with YNPE, they all showed the capability to compress λDNA, but with different DNA compaction dynamics from adding these proteins in YNPE*_hmo1Δ_*. When 8.2 nM of purified Hmo1 was directly pumped into the flow cell, the DNA molecule was compressed rapidly with faster dynamics than that observed in YNPE*_hmo1Δ_* supplemented with 8.2 nM Hmo1 (Fig. 2B, blue curve, and Fig. 2C). Interestingly, when introducing 8.2 nM of purified Hho1 directly into the flow cell, the DNA molecule was retracted to a dot within 60 seconds, although adding Hho1 in YNPE*_hmo1Δ_* to the final concentration of 8.2 nM showed no effect on DNA compaction for a long time (compare Fig. 2A and B, red curves, and Fig. 2C). These observations provided additional evidence that the DNA compaction observed in YNPE*_hmo1Δ_* by adding Hmo1 was not due to the simple protein-DNA interaction, protein aggregation, or DNA elasticity change. The chromatin assembly in YNPE seems to be a tightly regulated process similar to what occurred *in vivo,* and Hmo1 directly participates in this process.

### Hmo1 promotes the transition of nucleosomal arrays to a more compact conformation

Next, we employed TIRFM to visualize the fluorescent nucleosomes bound to the individual λDNA molecules in Hmo1-deficient YNPE. In budding yeast, the core histone H2B is encoded by two genes, *HTB1* and *HTB2*. We tagged these two genes with 3×FLAG tags at the *C*-terminus on their native chromosomal loci in the *hmo1Δ* strain to construct the *hmo1Δ*-H2B-3FLAG strain and extract its YNPE (Fig. S3A). On injection of the YNPE*_hmo1Δ-_*_H2B-3FLAG_ into the flow cell, the length of λDNA molecules reduced to 70% of the original length and remained unchanged, in line with what we observed before. We then labeled the nucleosomes *in situ* with fluorescent anti-FLAG antibodies and visualized them on TIRFM. By alternate excitation with 532 nm and 640 nm lasers, the fluorescent signals of nucleosomes on DNA were imaged. As the fluid flows, the fluorescent nucleosome can be observed to fluctuate along with the dsDNA. We observed one to four fluorescent signals on a single DNA molecule (Fig. 2D; Fig. S3B). The majority of DNA contained one or two fluorescent signals (accounting for 64% and 22%, respectively), while only a small percentage of DNA (10% and 4%) contained three or four fluorescent signals. Unfortunately, due to resolution limitations, we could not determine the exact number of nucleosomes in each fluorescent signal. Time courses confirmed that ≥99% of the fluorescent nucleosomes remained stably bound to the DNA for up to 20 minutes without dissociating or moving along the DNA. Furthermore, most fluorescent signals remained stably bound to DNA after washing with 0.5 to 1 M NaCl, ruling out the possibility of nonspecific bound florescent H2B (Fig. S3C). Subsequently, we supplemented YNPE*_hmo1Δ-_*_H2B-3FLAG_ with 8.2 nM Hmo1. As shown in Fig. 2D, λDNA was further compressed into a dot where the fluorescence of DNA and nucleosomes were co-localized. These results suggested that Hmo1 assisted the nucleosomal array in condensing into a more compact structure (Fig. 2D). In other words, the observations implied that the nucleosomes could not further fold into a higher compact form in the absence of Hmo1. It is well known that facilitating nucleosomes to compact into a higher-order structure is an exact function of linker histone H1 in metazoan (60).

### Single-molecule force spectroscopy suggests that Hmo1 facilitates nucleosome assembly in YNPE

**Fig 3:**
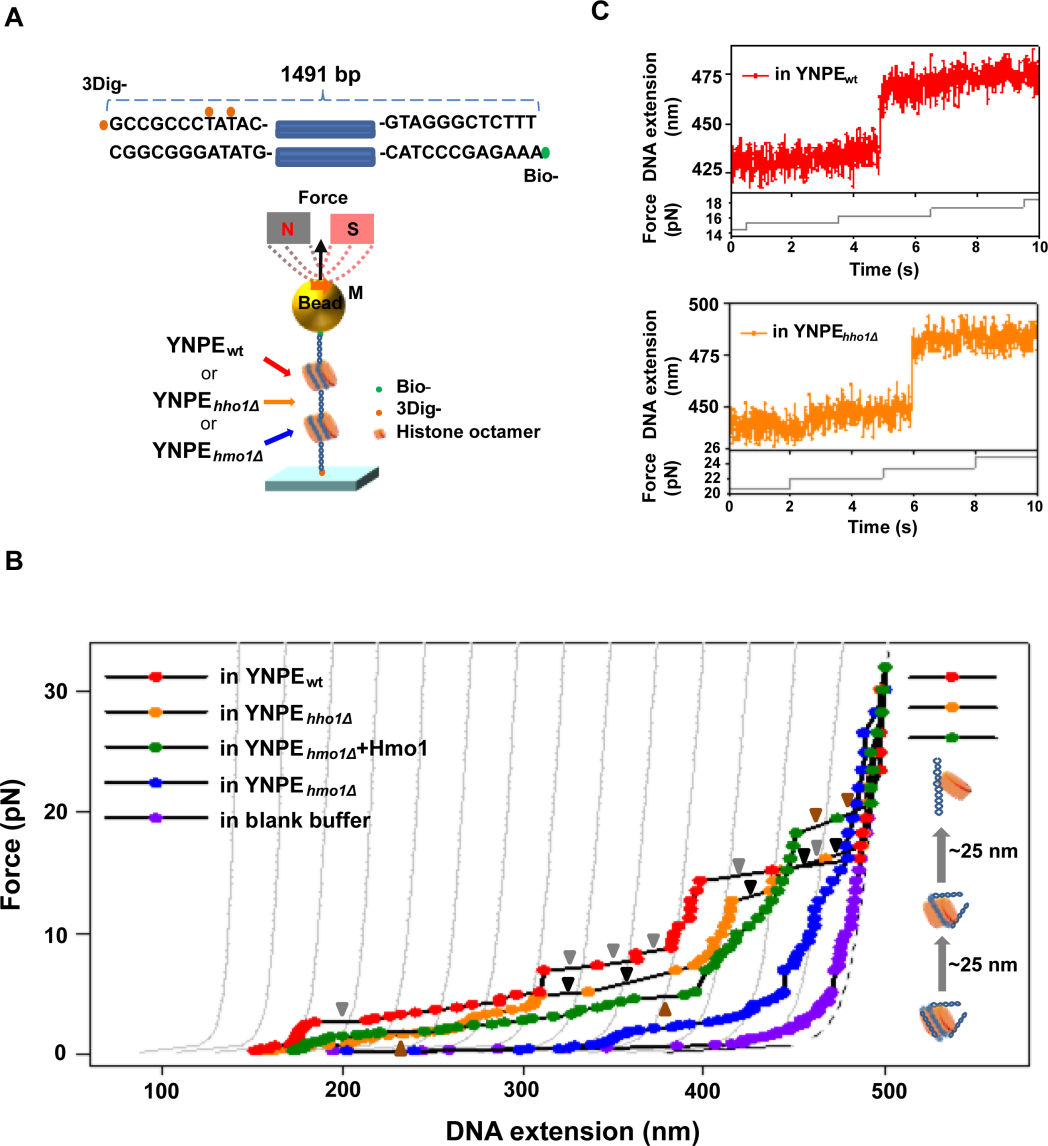
Single-molecule force spectroscopy demonstrated that Hmo1 facilitates the assembly of nucleosomes in YNPE. (**A**) Top: constructs of the DNA template: a 1,491-bp double-stranded DNA labeled with three digoxigenin (3Dig-) and biotin (Bio-) at each end. Bottom: schematic setup of the single-molecule magnetic tweezers used in our experiments. (**B**) The representative force-extension curves of chromatin assembled in different types of YNPE [YNPE_wt_ (red), YNPE*_hho1Δ_* (orange), YNPE*_hmo1Δ_* + Hmo1 (olive), YNPE*_hmo1Δ_* (blue)]. The control force-extension curve of 1,500-bp dsDNA was plotted in violet. All measurements were repeated more than five times. The WLC model fits the gray lines with different DNA contour lengths. The interval between two adjacent gray lines is 25 nm, representing half of the nucleosome dissociation length. Different colored triangles indicate the region where the jumps occurred in YNPE_wt_ (gray triangles), YNPE*_hho1Δ_* (black triangles), and YNPE*_hmo1Δ_* + Hmo1 (brown triangles), respectively. The right inset model depicts the nucleosome disruption process in YNPE_wt_ (red), YNPE*_hho1Δ_* (orange), and YNPE*_hmo1Δ_* + Hmo1 (olive). (**C**) Plots show the details of the representative jump during chromatin stretching in YNPE_wt_ (red) or YNPE*_hho1Δ_* (orange), indicating the dissociation of the nucleosomal wrap. The gray lines show the changes in force during the experiment.

To further investigate the role of Hmo1 in chromatin assembly in YNPE, we adopted single-molecule magnetic tweezers experiments. A 1,491-bp dsDNA substrate was designed and labeled with three digoxigenin molecules and biotin, and then attached to the flow cell surface by one end. YNPE_wt_, YNPE*_hho1Δ_*, and YNPE*_hmo1Δ_* diluted in blocking buffer were separately incubated with dsDNA for about 20 minutes at room temperature at extremely low force (<0.1 pN) to reconstitute chromatin (Fig. 3A). After the magnetic beads were attached, we stretched the chromatin formed in various YNPE with a continuously increasing force to track the conformational changes. During the stepwise disassembly events, we observed the disruption length of approximately 25 nm in both YNPE_wt_ (Fig. 3B, red curve) and YNPE*_hho1Δ_* (Fig. 3B, orange curve) in a single disruption event (Fig. 3B and C). The jump of 25 nm (corresponding to ~73.5 base pairs) is a typical event during nucleosome disassembly under mechanical tension, which indicates the unraveling of one DNA wrap from the histone octamer (61 - 65). In the force-extension curves of YNPE_wt_ and YNPE*_hho1Δ_*, a jump of integer multiples of 25 nm, such as 50 nm (~147 bp) or 75 nm (~220.5 bp) or 100 nm (~294 bp), occasionally occurred in one single disruption event, indicating the simultaneous rupture of several nucleosomal DNA wraps (Fig. 3B; Fig. S4A). Up to 3–4 nucleosomes were observed assembled on 1,500-bp dsDNA by YNPE_wt_ or YNPE*_hho1Δ_*, which is approximately half of the nucleosomes that can theoretically be accommodated on the 1,500-bp dsDNA (~up to 9 nucleosomes). The distribution of forces involved in the observed jumps was very broad (Fig. S4B). In contrast, no such extension jumps were observed in YNPE*_hmo1Δ_* (Fig. 3B, blue curve) throughout the entire stretching process. However, its force-extension curve deviated from the curve of bare DNA (Fig. 3B, blue curve and violet curve). This observed phenomenon could be attributed to no canonically assembled nucleosomes on the 1,500-bp dsDNA in Hmo1-deficient YNPE. Despite this, various proteins, including histones, might electrostatically interact with the dsDNA, causing the resulting curve (Fig. 3B, blue curve) to deviate from that of bare dsDNA (Fig. 3B, violet curve). When the force exceeds 30 pN, all the force-extension curves approached that of naked 1,500-bp dsDNA and that of dsDNA fitted by the worm-like chain (WLC) model (Fig. 3B, violet curve and black dashed curve), implying that all dsDNA unwrapped the histones.

To further verify the role of Hmo1 in chromatin assembly, we performed the magnetic tweezers experiments in YNPE*_hmo1Δ_* by supplementing with purified Hmo1 and H1.4, respectively. As shown in Fig. 3B; Fig. S5, supplementing Hmo1 or H1.4 in YNPE*_hmo1Δ_* resulted in force-extension curves resembling that of wild-type YNPE. The jumps representing the disruption of nucleosome wraps were clearly observed in force-extension curves (Fig. 3B, olive curve, and Fig. S5, dark yellow curve), implying the supplementation of Hmo1 or H1.4 effectively restored the nucleosome assembly in YNPE*_hmo1Δ_*, comparable to what occurred in the wild-type YNPE. In a single disruption event, the disruption length in YNPE was usually 25 nm (~73.5 bp) or multiples thereof, which indicated the rupture of the nucleosomal DNA wraps. However, the jumps formed by the single purified protein were variable and much larger in size (200 to 300 nm, corresponding to ~588–882 bp) and usually occurred only once at low forces (Fig. S4 and S6). The control experiments revealed distinct differences between unraveling the nucleosome formed by YNPE and disrupting the DNA loop formed by purified protein. This is consistent with our previous observations in the single-molecule fluorescence experiments (Fig. 2). In conclusion, combined with the observations on TIRFM before, it suggested that Hmo1 can facilitate nucleosome assembly in the YNPE system.

### Lysine-rich extension is essential for the chromatin assembly function of Hmo1

**Fig 4:**
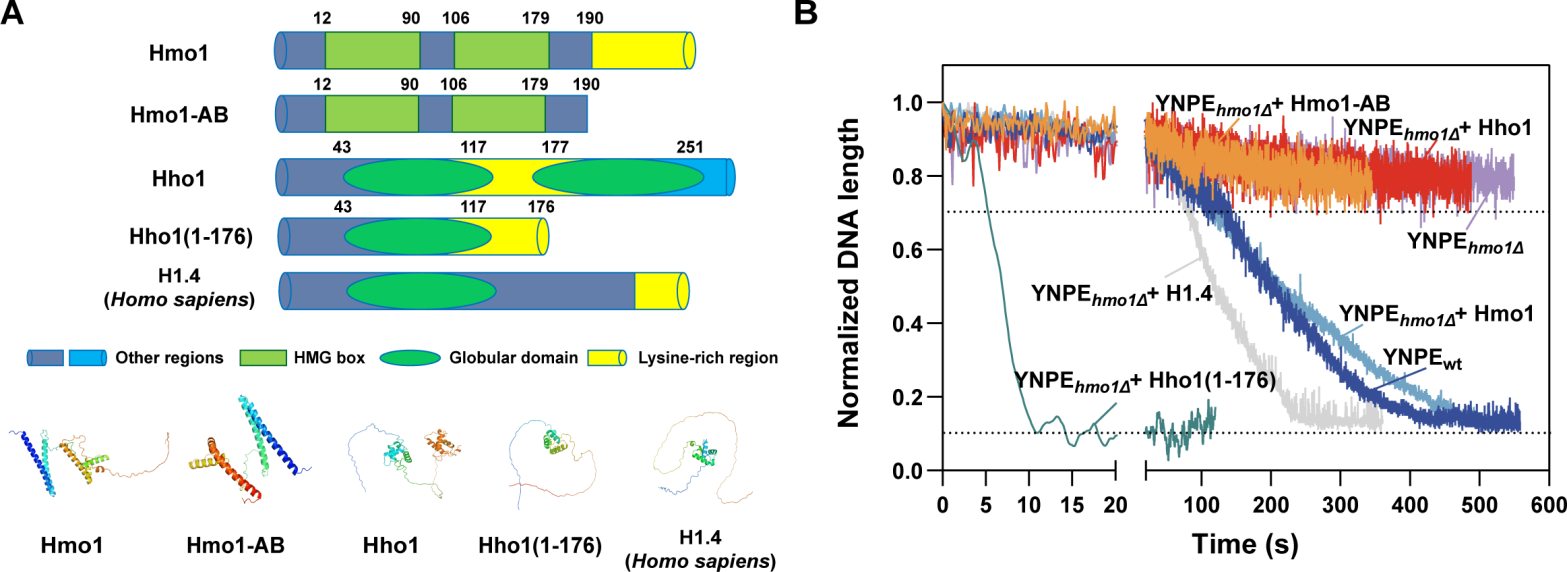
Lysine-rich extension is critical for the chromatin assembly function of Hmo1. (**A**) Top: schematic diagrams of Hmo1, Hmo1-AB, Hho1, Hho1(1-176), and H1.4(*Homo sapiens*). Hmo1-AB was derived by removing the portion of Hmo1 after the 190th amino acid, while Hho1(1-176) was derived by removing the portion of Hho1 after the 176th amino acid. Bottom: protein structure simulation of Hmo1, Hmo1-AB, Hho1, Hho1(1-176), and H1.4(*Homo sapiens*) by AlphaFold2 (Alphafold protein structure database, https://alphafold.ebi.ac.uk/). (**B**) Kinetic analysis of DNA compaction after supplementing the same concentration (8.2 nM) of purified Hmo1 (light blue), Hmo1-AB (orange), Hho1 (red), Hho1(1-176) (olive), and H1.4 (gray) in YNPE*_hmo1Δ_*. The curves of wild-type YNPE (dark blue) and the YNPE*_hmo1Δ_* (purple) were used as controls. More than 20 DNA molecules were measured in each experiment.

For human linker histone H1, it has been reported that the lysine-rich CTD plays an essential role in chromatin binding and condensation *in vivo* (20, 66, 67). Amino acid sequence analysis showed that both human H1.4 and Hmo1 possess a lysine-rich CTD. On the contrary, the lysine-rich region of Hho1 is sandwiched between its two globular domains (Fig. 4A). To determine the possible function of the lysine-rich region in chromatin assembly, Hmo1-AB, a truncated mutant of Hmo1 which lacked the lysine-rich CTD, was expressed and purified. Meanwhile, we also expressed and purified a Hho1 mutant, Hho1(1-176), in which the second globular domain was deleted, and the lysine-rich region turned to be the *C*-terminus (Fig. 4A). Unlike Hmo1, when 8.2 nM Hmo1-AB was introduced, chromatin assembly was not restored in YNPE*_hmo1Δ_*. The length of the λDNA molecules remained at 70% of the original length within 300 seconds, which was similar to the results of YNPE*_hmo1Δ_* and YNPE*_hmo1Δ_* + Hho1 (Fig. 4B). Interestingly, when 8.2 nM Hho1(1-176) was supplemented in YNPE*_hmo1Δ_*, the λDNA molecules were compacted from 70% of the original length to a dot at a much faster rate (about 10 seconds) (Fig. 4B). The results indicated that the lysine-rich CTD is required for the chromatin assembly function of Hmo1. For Hho1, it seems that the second globular domain buries the lysine-rich region so as to hinder its function as linker histone H1.

### Hmo1 possesses phase separation properties when mixed with dsDNA

Linker histone H1 regulates the dynamic organization of chromatin by promoting liquid-liquid phase separation (LLPS), and its highly disordered lysine-rich CTD is essential for this process (18, 19, 33, 68). Since Hmo1 possesses a similarly long lysine-rich CTD which was predicted to be disordered (Fig. S7A), we examined whether Hmo1 also induced LLPS.

**Fig 5:**
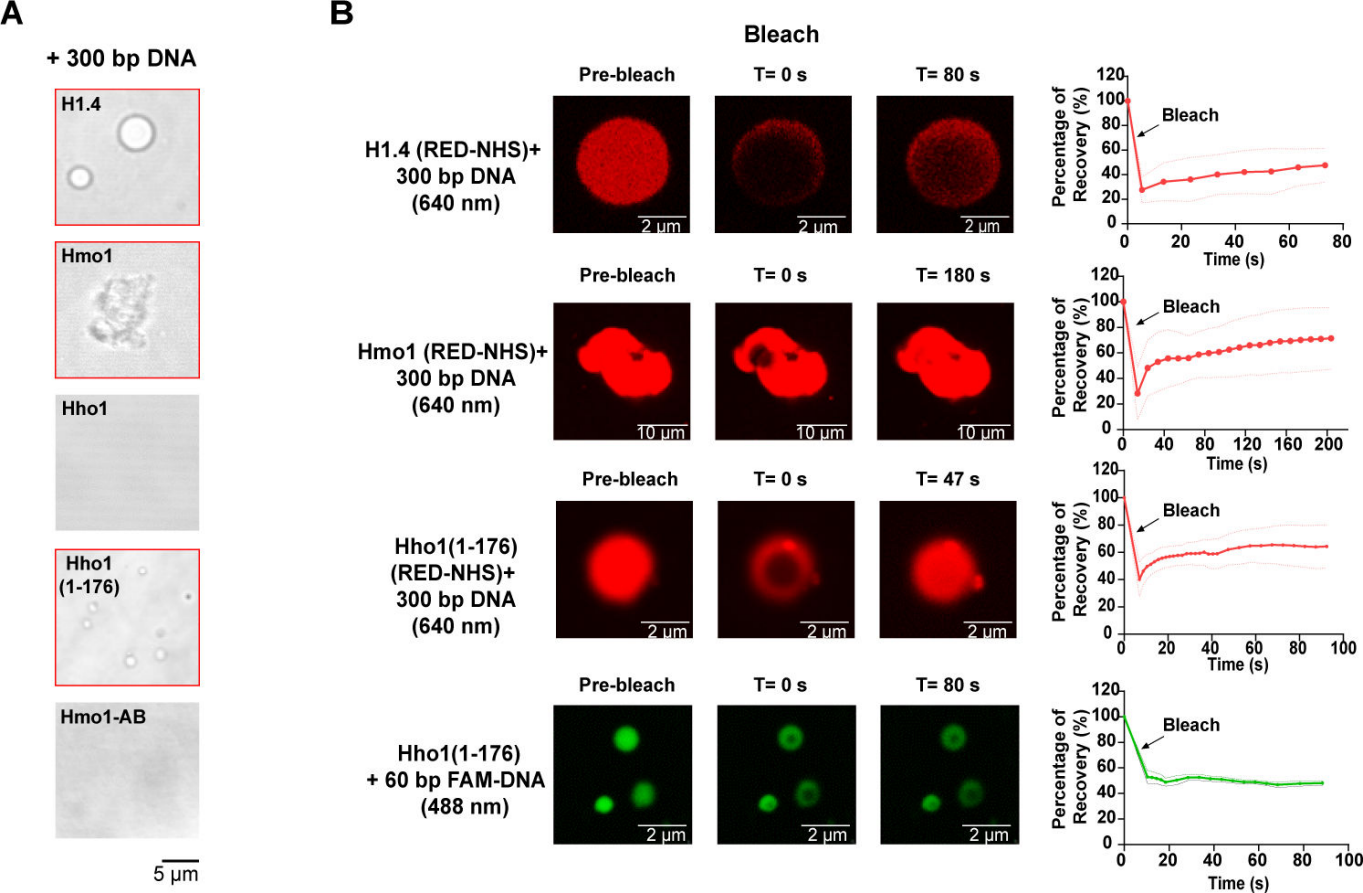
Hmo1 possesses phase separation property when mixed with dsDNA. (**A**) Bright-field images of five kinds of purified proteins mixed with 300-bp DNA. The images with red borders represent the occurrence of phase separation. (**B**) Snapshot images of phase separation condensate before and after photobleaching and recovery (left). Purified proteins [H1.4, Hmo1, Hho1(1-176)] were labeled with red dyes carrying the NHS-ester group (RED-NHS). 60-bp dsDNA was labeled with fluorescein amidite (FAM). The percentage of recovery of phase separation condensates versus time (right; mean ± SD, *n* = 3).

Similar to human linker histone H1.4, Hmo1 alone did not form condensates. When mixed with 300-bp dsDNA, the Hmo1-DNA mixture readily formed many condensates. Unlike the spherical droplets formed by H1.4-DNA, the condensates formed by Hmo1-DNA were irregular and gel-like. The condensates were dissolved in the presence of high salt concentration, showing a reversible character (Fig. 5A; Fig. S7B). The phenomenon is similar to the coarse condensates formed by H1-polynucleosomes (33). In contrast, Hho1 did not form a condensate even when mixed with dsDNA (Fig. 5A). To determine the mobility of Hmo1 inside the irregular condensates, we monitored the fluorescence recovery after photobleaching (FRAP) (69). In the Hmo1-DNA condensate, significant fluorescence recovery was observed shortly after photobleaching, indicating that Hmo1 was mobile within the condensates (Fig. 5B). However, unlike the droplets formed by H1.4-DNA, which disappeared with the increase of the DNA length, the Hmo1-DNA condensates appeared nearly unaffected by DNA length (Fig. S7B).

Meanwhile, we tested the two mutants, Hmo1-AB and Hho1(1-176), for phase separation. Hmo1-AB, which lacked the disordered *C*-terminus, failed to form condensates in the presence of dsDNA. However, Hho1(1-176) readily undergone LLPS when mixed with 300-bp dsDNA, similar to the observations in the H1.4-DNA mixture. FRAP experiments showed a highly dynamic diffusion of Hho1(1-176) within the spherical droplet (Fig. 5A and B). Interestingly, as shown in Fig. S7B, the morphology of the Hho1(1-176)-DNA condensates gradually changed from the initial spherical droplets to large gels as the DNA length increased. After adding a certain amount of DNase, the large gel-like condensates reverted to droplets again (Fig. S7B). Meanwhile, we used the FAM-labeled dsDNA to examine whether DNA was dynamically diffused in the condensates. As shown in Fig. 5B, the bleached DNA did not undergo recovery in the droplet, suggesting that the dsDNA might have been arrested in it.

### Fluctuation in Hmo1 phosphorylation coincides with linker histone H1 during the cell cycle

Linker histone H1 is phosphorylated in a cell cycle-dependent manner and correlates with the open or closed states of chromatin. The extent of H1 phosphorylation progressively increases as the cell progresses from G1 to mitosis and then sharply decreases. Low phosphorylation levels of H1 in the S-phase are associated with an open chromatin conformation that facilitates replication and transcription events. In M-phase, high phosphorylation levels of H1 promote the entry of condensins (7, 15, 70). Given that many properties of Hmo1 are similar to those of H1, we sought to detect the phosphorylation of Hmo1 during the cell cycle.

**Fig 6:**
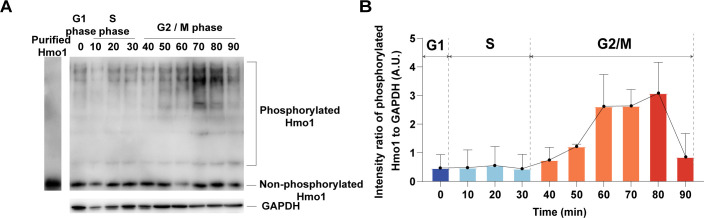
The degree of phosphorylation of Hmo1 changes during the cell cycle in a manner consistent with that of H1. (**A**) The representative western blotting image of Hmo1 phosphorylation. The experiments were performed three times. (**B**) Relatively quantification of western blotting data (mean ± SD, *n* = 3). The degree of Hmo1 phosphorylation varies across the cell cycle. The *y*-axis represents the intensity ratio of phosphorylated Hmo1 to GAPDH.

The yeast cells were released from the synchronization at the G1 phase and harvested cells every 10 minutes to extract the whole cell proteins. The cell cycle phase of harvested cells was determined by flow cytometry analysis (Fig. S8). As shown in Fig. 6A and B, the extent of Hmo1 phosphorylation was variable during the cell cycle. At different periods of the cell, Hmo1 was usually present as a mixture of unphosphorylated and phosphorylated forms. The phosphorylation level of Hmo1 was lowest in G1 phase and increased during the S and G2 phases. The highest phosphorylation level was determined at the end of the G2 phase and mitosis when the expression of *HMO1* reached the highest level (Fig. 6A and B). Accordingly, the phosphorylation extent of Hmo1 at different cell cycle stages is consistent with that of linker histone H1. This observation is in agreement with the hypothesis that Hmo1 might function as a linker histone to regulate chromatin structure in *S. cerevisiae* (22, 23).

## DISCUSSION

The identity of the histone H1 protein in budding yeast, whether it is Hho1 or Hmo1, has been long debated. In this study, we utilized the single-molecule approach to directly investigate the chromatin assembly in an *in vitro* biochemical system (YNPE), which can accurately simulate the physiological conditions in the nucleus. The observations revealed that Hmo1, rather than Hho1, plays a crucial role in promoting DNA compaction and facilitating chromatin assembly. Furthermore, we employed magnetic tweezers to demonstrate that Hmo1 can facilitate the formation of nucleosomes in YNPE from the mechanical aspect. Same as metazoan histone H1, Hmo1 exhibits phase separation properties when mixed with dsDNA. Moreover, the extent of Hmo1 phosphorylation at various cell cycle stages is similar to that of linker histone H1. These findings have implications that Hmo1 might take some functions of histone H1 in budding yeast.

Unlike higher eukaryotes, budding yeast has a unique chromatin structure, such as small linker DNA, atypical heterochromatin, and highly acetylated core histones (71 - 74). The more open chromatin conformation may allow the yeast to perform transcriptional events at a fast rate (75, 76). Therefore, yeast has long been thought to lack linker histone H1 (77). Hho1 was proposed to be histone H1 of budding yeast based on the amino acid sequence similarity alignment (26). However, the chromatin structure and the sensitivity to nuclease were not altered in the *hho1Δ* strain (25, 34). In this study, we demonstrated the deletion of *HHO1* did not affect the chromatin assembly in YNPE (Fig. 1, Fig. 2A, and Fig. 3). It is likely that Hho1 might not function as a linker histone in *S. cerevisiae*. On the contrary, the observations in single-molecule experiments suggested that Hmo1 is essential for chromatin assembly. Hmo1 is a unique member of the HMGB protein family, which showed the ability to maintain chromatin integrity in genetic studies (22, 39, 48, 78). In the YNPE derived from the *hmo1Δ* strain, λDNA was only compacted to 70% of its original length and was not further compressed. When supplementing Hmo1 in YNPE*_hmo1Δ_*, the compaction of λDNA was restored (Fig. 1; Fig. 2). A similar phenomenon was observed for supplementing the histone H1.4 but not for bacterial HU protein (Fig. 2A). In the YNPE*_hmo1Δ_*, only a few discrete distributions of fluorescent nucleosomes were observed on λDNA, and no nucleosome was successfully assembled on 1.5 k dsDNA in magnetic tweezers experiments (Fig. 2D; Fig. 3B). Supplementation of purified Hmo1 rendered the nucleosomal chain into a more compacted structure, and the nucleosome assembled on the 1.5 k dsDNA (Fig. 2D; Fig. 3B, olive curve). Based on the observations from TIRFM and magnetic tweezers experiments, it is unlikely that this process was simply due to the DNA-looping function of Hmo1 and H1.4 since the DNA-looping protein HU did not restore the further DNA compaction in YNPE*_hmo1Δ_*, and there were markable differences between the DNA force-extension curves with or without YNPE (Fig. 2 and 3; Fig. S6). Our data provide evidence that Hmo1, but not Hho1, facilitates chromatin assembly in budding yeast.

However, the detailed molecular mechanism of how Hmo1 facilities the chromatin assembly still needs to be discovered. It has been proposed that Hmo1 bound near the dyad axis with its box A; thus, the lysine-rich CTD interacted with linker DNA to form a stem-like structure to prevent DNA bending, similar to linker histone H1 (23). But some studies suggested that Hmo1 strongly bound to the nucleosome and unwound DNA from the core nucleosome to separate linker DNA, especially at a low protein concentration (24, 79). These observations against the function of Hmo1 acting as histone H1 and explained the function of Hmo1 as an HMGB protein to promote transcription. Interestingly, at a high protein concentration (>3 nM), the presence of Hmo1 significantly compacted nucleosome arrays and shortened nearest neighbor nucleosome spacing (79). In this study, we supplemented the YNPE*_hmo1Δ_* with 8.2 nM Hmo1 and observed the restoration of DNA compaction (Fig. 2A). It was estimated that the average concentration of Hmo1 was 12.5 µM in yeast nuclei (42, 80, 81). In the real chromatin assembly process, the local concentration of Hmo1 around the core nucleosome could be even higher. Accordingly, we hypothesized that Hmo1 might bind to the nucleosomes and link adjacent nucleosomes via oligomerization. This hypothesis is supported by the fact that Hmo1 can form higher-order oligomers (82, 83) and shows gel-like phase separation properties when mixed with dsDNA (Fig. 5).

We demonstrated that the chromatin assembly function of Hmo1 depended on its lysine-rich CTD (Fig. 4B). Unlike the other HMGB proteins, Hmo1 possesses a unique lysine-rich CTD similar to metazoan histone H1. Hho1 also contains a lysine-rich region, but the lysine-rich region is buried between two globular domains (Fig. 4A). Deletion of the second globular domain exposes the lysine-rich region. Interestingly, similar to histone H1 or Hmo1, the Hho1 mutant (1-176) restored the DNA compaction in YNPE*_hmo1Δ_* and showed the phase separation property. It is possible that the budding yeast Hho1 accidentally acquired an extra globular domain by recombination during the evolution, which resulted in the loss of its chromatin assembly function. In this case, the HMGB family protein Hmo1 might perform some of the linker histone functions instead. Certainly, we cannot rule out another possibility that the metazoan histone H1 evolved from the Hho1 by losing the globular domain at the *C*-terminus.

Currently, accumulating evidence favors the fluid-like model of chromatin structure (18, 84). The linker histone H1 has been proven to promote phase separation of chromatin, which is consistent with its role in promoting chromatin condensation in cells (18). Similar to histone H1, Hmo1 showed the phase separation property when incubated with dsDNA. We found that the lysine-rich CTD of Hmo1 was necessary for forming condensates, which further demonstrated the importance of lysine-rich CTD for chromatin assembly. Unlike the spherical droplets formed by human linker histone H1.4, Hmo1 formed gel-like condensates with dsDNA. This phenomenon indicated stronger Hmo1-DNA and Hmo1-Hmo1 interactions (Fig. 5). In addition, Hmo1 was phosphorylated in the same fluctuation as H1 in the cell cycle, suggesting the regulation of Hmo1 activity by the post-translational modifications during the cell cycle (Fig. 6). Collectively, we speculated that Hmo1 could perform some functions as histone H1 to facilitate the chromatin assembly, potentially utilizing its lysine-rich CTD that participates in phase separation.

The established model for linker histone H1’s function in packaging nucleosomal array into a higher-order chromatin structure is primarily based on purified proteins or purified chromatin studies *in vitro* (85 - 88). However, the role of H1 *in vivo* is less certain. YNPE is a robust biochemical system that mimics the physiological conditions *in vivo* to some extent. This study showed the biochemical characteristics of the purified proteins (H1.4, Hmo1, Hho1, and HU) *in vitro* differ from those shown in YNPE (Fig. 2 and 3; Fig. S5 and S6), suggesting that a protein’s contribution to chromatin assembly is strongly regulated by multiple other proteins *in vivo*, rather than relying solely on its biochemical properties. This may explain some discrepancies in behavior observed between purified Hmo1 (24, 79) and Hmo1 in YNPE. In the future, ultra-resolution imaging techniques and highly sensitive mechanical measurements appropriate for use in YNPE are highly desirable to obtain a more comprehensive understanding of the biochemical details of chromatin assembly that occur *in vivo*.

## MATERIALS AND METHODS

### Yeast strains, plasmids construction, and proteins

The *hmo1Δ* strain, *hho1Δ* strain, *hmo1Δ & hho1Δ* strain were constructed in W303-10A (MATa *ade-1 can1-100 his3-11*, *15 leu2-3*, *112 trp1-1 ura3-1*) background. The *hmo1Δ*-H2B-3FLAG strain was constructed by labeling *HTB1* and *HTB2* genes with 3×FLAG at their native loci in the chromosome of the *hmo1Δ* strain using the CRISPR-Cas9 method.

DNA fragments encoding Hmo1, Hho1, H1.4, Hho1(1-176), and Hmo1-AB (1-190) were inserted into the plasmid vector pET28a to construct various plasmids for expressing the corresponding proteins. To label the core histone H2B, two plasmids, pWYE3240-*HTB1*sgRNA-Leu and pWYE3240-*HTB2*sgRNA-Trp, were constructed by homologous recombination. The optimal gRNA sequences were designed by the Cas-Designer website CRISPR RGEN Tools (rgenome.net).

All proteins were expressed in *E. coli* BL21 (Biomed Inc.) with minor modifications (89). For example, Hmo1 was purified by a His-Trap HP column (binding buffer: 20 mM Tris-HCI pH 7.5, 500 mM NaCl; elution buffer: 20 mM Tris-HCI pH 7.5, 500 mM NaCl, 500 mM imidazole) and a Hi-Trap SP HP column (binding buffer: 20 mM Tris-HCI pH 6.5, 200 mM NaCl; elution buffer: 20 mM Tris-HCI pH 6.5, 1 M NaCl) using the AKTA purifier. The Anti-Hmo1 antibody was purchased from Abcam (ab71834), the Anti-GAPDH antibody was purchased from ABclonal (AC033), the secondary antibody Goat-Anti-Mouse IgG (H&L) HRP and Goat-Anti-Rabbit IgG (H&L) HRP were purchased from Abmart (M21001L and M212115), and the Anti-FLAG antibody was purchased from Abbkine (A02010A680).

### Preparation of the YNPE and single-molecule visualization with total internal reflection fluorescence microscopy (TIRFM)

YNPEs from different yeast strains were prepared as described by Chen et al. (56). The protein concentrations of YNPEs were measured using the Bradford Protein Concentration Assay Kit (Beyotime Inc.). Single-molecule experiments were performed in the flow cell. Flow cell assembly and λDNA biotinylation were modified from our existing procedure (90). Streptavidin of 10 µL (0.5 mg/mL, Sigma-Aldrich) was injected into the flow cell and incubated for 2–3 minutes at room temperature. After the outlet tubing was connected to the aspiration syringe pump (Harvard Instruments, standard injection/extraction PHD ULTRA), the flow cell was flushed with blocking buffer [20 mM Tris-HCl pH 7.5, 50 mM NaCl, 2 mM EDTA, 0.2 mg/mL bovine serum albumin (BSA)] at a rate of 100 µL/min. Next, diluted biotin-λDNA (final concentration: 1.5 nM) containing 10 nM Sytox orange (Thermo Fisher Scientific) was pumped into the flow cell at 50 µL/min for approximately 30 seconds. Excess DNA was washed with the blocking buffer containing Sytox dye at 100 µL/min. After immobilizing λDNA molecules at one end in the flow cell, the flow cell was washed with buffer, and YNPE was introduced at the rate of 50 µL/min. The inverted TIRFM [modified from IX-71 Olympus, 100× objective with numerical aperture (N.A.) = 1.49] equipped with an Andor iXON ultra 897 EMCCD was used to acquire the images of DNA molecules under excitation with a 532-nm laser. Videos recording DNA length changes were captured by an electron multiplying charge coupled device (EMCCD) with 0.1-second exposure and 0.1-second interval for 10 minutes. The imaging data were analyzed as previously described (56).

### Observation of fluorescently labeled nucleosomes on DNA

The procedure described by Visnapuu and Greene was followed with modifications to label the nucleosomes with fluorescent dyes (91). Briefly, the YNPE*_hmo1Δ_*_-H2B-3FLAG_ was diluted with the blocking buffer containing 10 nM Sytox orange and pumped into the flow cell at 50 µL/min until the DNA length no longer changed. The anti-FLAG tag antibody (Abbkine) of 2 µg/mL was pumped into the flow cell at 50 µL/min and incubated for 10 minutes. After washing the unbound antibody with the blocking buffer, the fluorescence of DNA and histone H2B were visualized under the excitation by 532 nm and 640 nm lasers. The co-localization of nucleosome fluorescence and DNA was determined by the fluctuation along with DNA in the fluid flow.

### Single-molecule mechanical assay with magnetic tweezers

DNA substrate with the random sequence was labeled with three digoxigenin molecules and biotin at each end. The flow chamber was assembled by two coverslips and functionalized with anti-digoxigenin protein. Reference beads were attached to the flow chamber for drift correction. In the magnetic tweezers assay, the same concentration (100 µg/mL) of different types of YNPE was first incubated with double-stranded DNA (dsDNA) for 20 minutes at room temperature to assemble chromatin, then the chromatin assembled in YNPE was bound to a functionalized coverslip at one end and to a magnetic bead (M280, Invitrogen) at the other end. As for the magnetic tweezers experiments with purified proteins, the same concentration (10 nM) of purified protein (Hmo1 or H1.4 or LmHU) was added to the flow chamber after the dsDNA bound to the magnetic bead and then incubated for 5 minutes. The blank buffer in the experiments referred to the blocking buffer (20 mM Tris-HCl pH 7.5, 50 mM NaCl, 2 mM EDTA, 0.2 mg/mL BSA), which was used as a dilution buffer for all YNPE and purified proteins. The magnetic bead moved along the Z-axis stepwise under the action of the changing external force, which was applied by moving the permanent magnet above the flow chamber at a rate of 62.5 µm/step (>1 pN, constant force with 3 seconds per step). The forces in the magnetic tweezers experiments varied from ~0.2 to ~40 pN. The diffraction ring shape of the magnetic bead was observed with a microscope (100×, N.A. 1.45; Olympus) to monitor the DNA extension (92). The magnetic bead was rotated 50–100 turns clockwise or counter-clockwise with a force of ~1–2 pN to check for the individually tethered dsDNA. After rotation, the magnetic beads with the invariant positions on the *z*-axis were selected to plot the force-extension curves. The curves fitted by the worm-like chain (WLC) model formula were used as a marker for DNA extension (93).

### Phase separation and fluorescence recovery after photobleaching (FRAP) assays

Protein concentrations were diluted to 10 µM with blocking buffer and further fluorescently labeled with the RED-NHS Protein Labeling Kit (Monolith). Protein and dsDNA were mixed at equal concentrations of 10 µM (final nucleic acid to protein ratio of approximately 1, N/P ~ 1) in a centrifuge tube and incubated for 2–3 minutes at room temperature. Then the mixture was gently transferred to a confocal petri dish (35 mm diameter; Biosharp Inc.). Phase separation was observed using a Leica SP8 laser confocal microscope (100×, N.A. 1.4) under the bright field, 633-nm or 488-nm laser. The 300-bp dsDNA substrate was obtained by PCR (primer 5′−3′: atgcaaagcccatatccaatgacac and gcctcttttacctcgagggtgg) from the yeast genome. FAM-labeled and unlabeled 60-bp dsDNA were synthesized by Tsingke Biotechnology. pBR322 and λDNA were purchased from TaKaRa and New England Biolabs. DNAase was purchased from YEASEN. FRAP assays were carried out as previously described by Taylor et al. (69). A self-selected circular region of interest (ROI) within the phase separation assembly was photobleached by laser excitation, and the recovery process was observed for approximately 3 minutes. Fluorescence intensity was normalized and plotted against a function of time.

### Phosphorylation detection of Hmo1

The yeast cells were synchronized in the G1 phase by arresting with α factor (5 µg/mL). The cell cycle phase was determined by microscopic cell morphology and flow cytometry (BD FACS Calibur). After being released from arrest, yeast cells were collected every 10 minutes to extract total proteins. Phosphorylation of Hmo1 was detected by the acrylamide-dependent Phos-tag kit (Wako). The protein samples from 0 to 90 minutes were electrophoresed and detected with the anti-Hmo1 antibody (ab71834) and the anti-GAPDH antibody (AC033).

## Data Availability

All relevant data are included in the manuscript and the Supplementary material file.

## SUPPLEMENTAL MATERIAL

Supplemental Figure Legends mbio.00993-23-s0001.docx Legends for Figs. S1 to S8.

Fig. S1 mbio.00993-23-s0002.tif Gaussian fits of the original length of λDNA and the kinetic analysis of DNA compaction by YNPEwt.

Fig. S2 mbio.00993-23-s0003.tif Kinetic analysis of DNA compaction in YNPE_*hmo1*Δ_ supplemented with different concentrations of purified Hmo1.

Fig. S3 mbio.00993-23-s0004.tif Fluorescent labeling of nucleosomes.

Fig. S4 mbio.00993-23-s0005.tif Histograms of step sizes (bp) and forces (pN) in disruption events during chromatin stretching in YNPEwt.

Fig. S5 mbio.00993-23-s0006.tif The representative force-extension curves of chromatin assembled in YNPE_*hmo1*Δ_+ H1.4.

Fig. S6 mbio.00993-23-s0007.tif The representative force-extension curves of dsDNA under different conditions: with purified Hmo1 or H1.4, or LmHU, and in the blank buffer.

Fig. S7 mbio.00993-23-s0008.tif Hmo1 has a multi-component phase separation property.

Fig. S8 mbio.00993-23-s0009.tif Analysis of the budding yeast cell cycle by flow cytometry.

ASM does not own the copyrights to Supplemental Material that may be linked to, or accessed through, an article. The authors have granted ASM a non-exclusive, world-wide license to publish the Supplemental Material files. Please contact the corresponding author directly for reuse.
