# Supplementary material for: Single-molecule study reveals Hmo1, not Hho1, promotes chromatin assembly in budding yeast
Source: mBio. 2023 Jul 11;14(4):e00993-23. doi: 10.1128/mbio.00993-23 (PMC10470511; doi:10.1128/mbio.00993-23)
Supplement: Supplemental Figure Legends — Legends for Figs. S1 to S8. [file mbio.00993-23-s0001.docx]

**SUPPLEMENTARY**

**FIG S1** Gaussian fits of the original length of λDNA and the kinetic analysis of DNA compaction by YNPE_wt_. (A) Histogram of the length of λDNA molecules before YNPE injection. The average length was calculated by Gaussian fit (300 DNA molecules in total). (B) Kinetic analysis of DNA compression by two diluted wild-type YNPE (1:200 YNPE_wt_ (Dark blue), 1:2000 YNPE_wt_ (Light Blue)). Each experiment measured more than 30 DNA molecules.

**FIG S2** Kinetic analysis of DNA compaction in YNPE*_hmo1Δ_* supplemented with different concentrations of purified Hmo1 (YNPE*_hmo1Δ_*+ 32.7 nM Hmo1 (Yellow), YNPE*_hmo1Δ_*+ 16.4 nM Hmo1 (Blue), YNPE*_hmo1Δ_*+ 8.2 nM Hmo1 (Light blue), YNPE*_hmo1Δ_*+ 4.1 nM Hmo1 (Green), YNPE*_hmo1Δ_* (Purple)). The dark blue wild-type YNPE curve was used as a control. More than 20 DNA molecules were measured in each experiment.

**FIG S3** Fluorescent labeling of nucleosomes. (A) The schematic diagram shows the locus of nucleosome H2B C-terminus. The structure of the nucleosome was derived from PDB (accession number:1ID3). (B) The statistical histogram illustrates the number of fluorescent signals representing nucleosomes binding on a single λDNA in YNPE*_hmo1Δ-_*_H2B-3FLAG_ (50 DNA molecules in total). (C) After washing with 1 M NaCl, the fluorescent signal remained bound to the dsDNA.

**FIG S4** Histograms of step sizes (bp) and forces (pN) in disruption events during chromatin stretching in YNPE_wt_. (A) Histogram of step sizes (bp) in disruption events during chromatin stretching in YNPE_wt_. The average lengths were calculated by Gaussian fit (n=70). (B) Histogram of the forces (pN) at which jumps occurred during the chromatin stretching in YNPE_wt_ (n=70).

**FIG S5** The representative force-extension curves of chromatin assembled in YNPE*_hmo1Δ_*+ H1.4 (Dark yellow), YNPE_wt_ (Red), and YNPE*_hmo1Δ_* (Blue). The force-extension curve of only 1500 bp dsDNA was plotted in violet. The black triangle indicates where the jumps occurred in YNPE*_hmo1Δ_*+ H1.4. All measurements were repeated more than five times. The Worm-like Chain (WLC) model fits the grey lines with different DNA contour lengths. The interval between two adjacent grey lines is 25 nm, representing half of the nucleosome dissociation length. The right inset model depicts the nucleosome disruption process in YNPE_wt_ (Red) and YNPE*_hmo1Δ_*+ H1.4 (Dark yellow).

**FIG S6** The representative force-extension curves of dsDNA under different conditions: with purified Hmo1 (Cyan) or H1.4 (Navy), or LmHU (Light green), and in the blank buffer (Violet). The grey, black or brown triangle indicates the disruption of the loop formed by Hmo1-dsDNA, H1.4-dsDNA, and LmHU-dsDNA, respectively. All measurements were repeated more than ten times. The Worm-like Chain (WLC) model fits the grey lines with different DNA contour lengths. The interval between two adjacent grey lines is 25 nm. The right inset model depicts the loop disruption in Hmo1-dsDNA, H1.4-dsDNA, or LmHU-dsDNA.

**FIG S7** Hmo1 has a multi-component phase separation property. (A) Prediction of the distribution of the disordered regions of four different proteins (H1.4, Hmo1, Hho1, and Hho1(1-176)) using PONDR (http://www.pondr.com/). (B) Bright-field images of proteins (from top to bottom: H1, Hmo1, Hho1(1-176)) mixed with different lengths of dsDNA (from left to right: no dsDNA, 60 bp dsDNA, 300 bp dsDNA, pBR322 DNA, λDNA). The last column showed the disappearance of condensates formed by Hmo1+300 bp dsDNA under high salt concentration and the morphological changes of the condensates formed by Hho1(1-176) +λDNA in the presence of nuclease. Images with red borders represent the occurrence of phase separation.

**FIG S8** Analysis of the budding yeast cell cycle by flow cytometry. The first peak corresponds to the G1 phase, the second peak corresponds to the S phase, and the third peak corresponds to the G2 phase. The red arrow points to the number 600 at the x-axis of the DNA content, and the green arrow points to the number 1200 at the x-axis of the DNA content.
