# Supplementary figures and images for: Single-molecule study reveals Hmo1, not Hho1, promotes chromatin assembly in budding yeast
Source: mBio. 2023 Jul 11;14(4):e00993-23. doi: 10.1128/mbio.00993-23 (PMC10470511; doi:10.1128/mbio.00993-23)

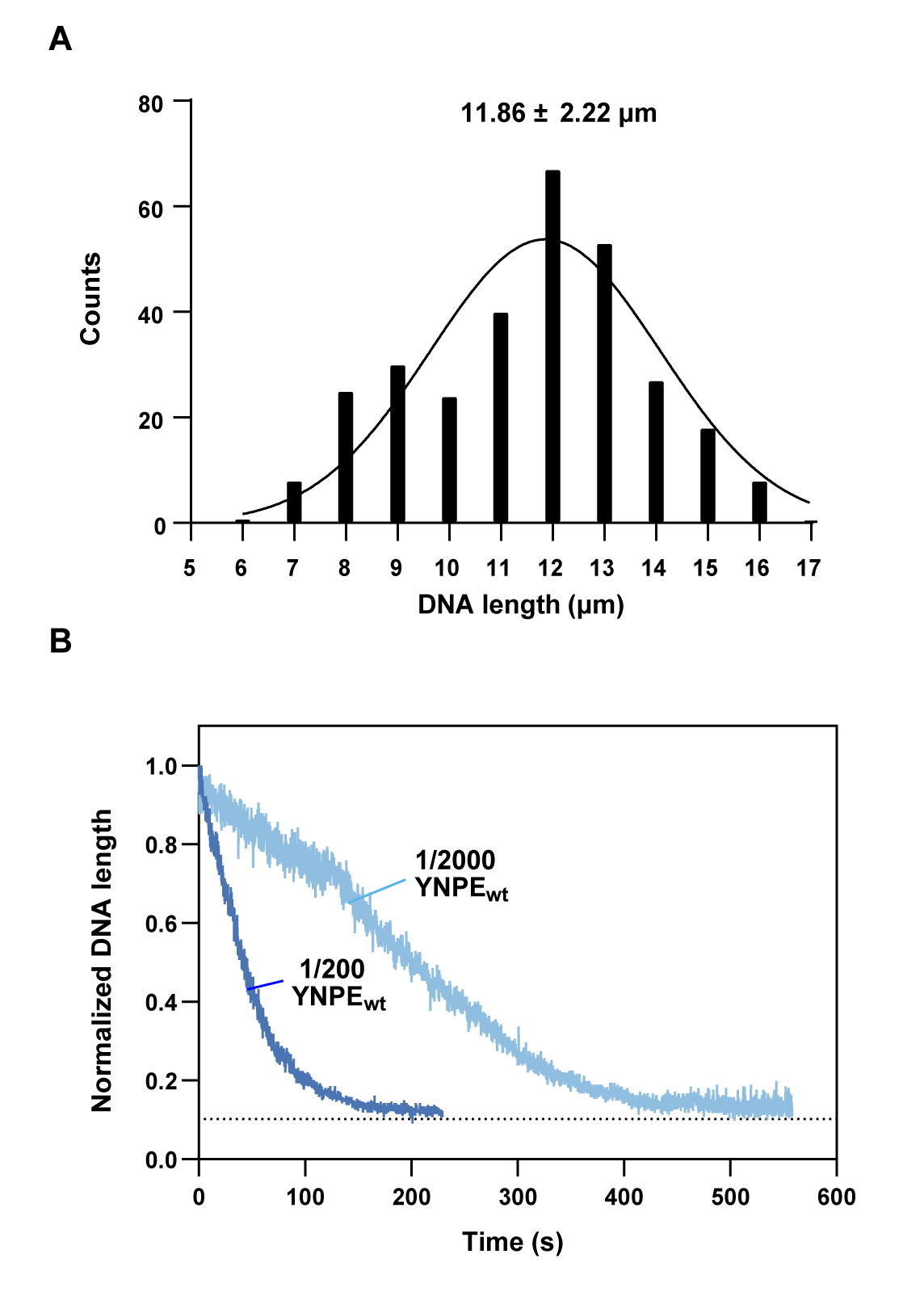

Supplement: Fig. S1 — Gaussian fits of the original length of λDNA and the kinetic analysis of DNA compaction by YNPEwt. [file mbio.00993-23-s0002.tif]

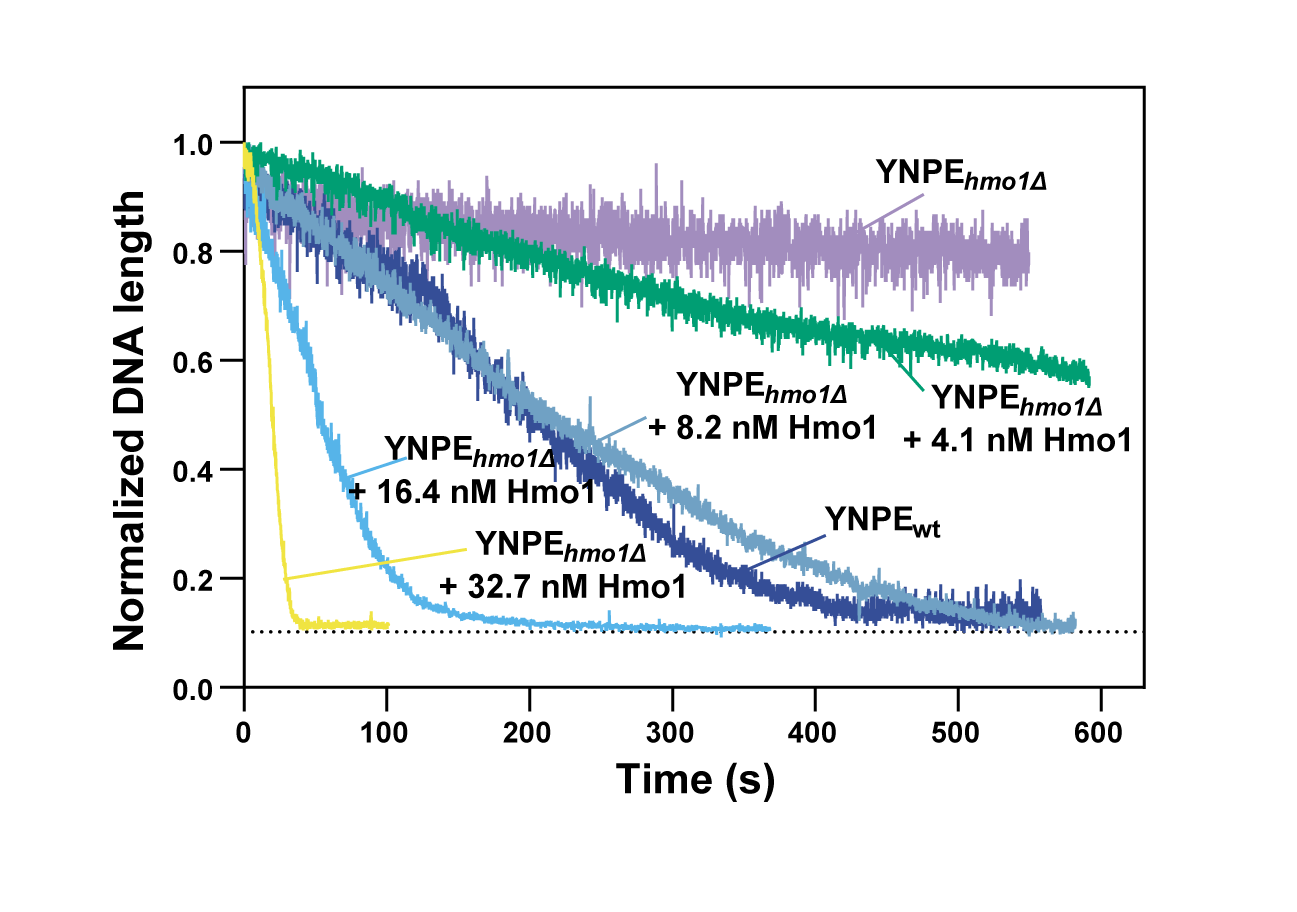

Supplement: Fig. S2 — Kinetic analysis of DNA compaction in YNPEhmo1Δ supplemented with different concentrations of purified Hmo1. [file mbio.00993-23-s0003.tif]

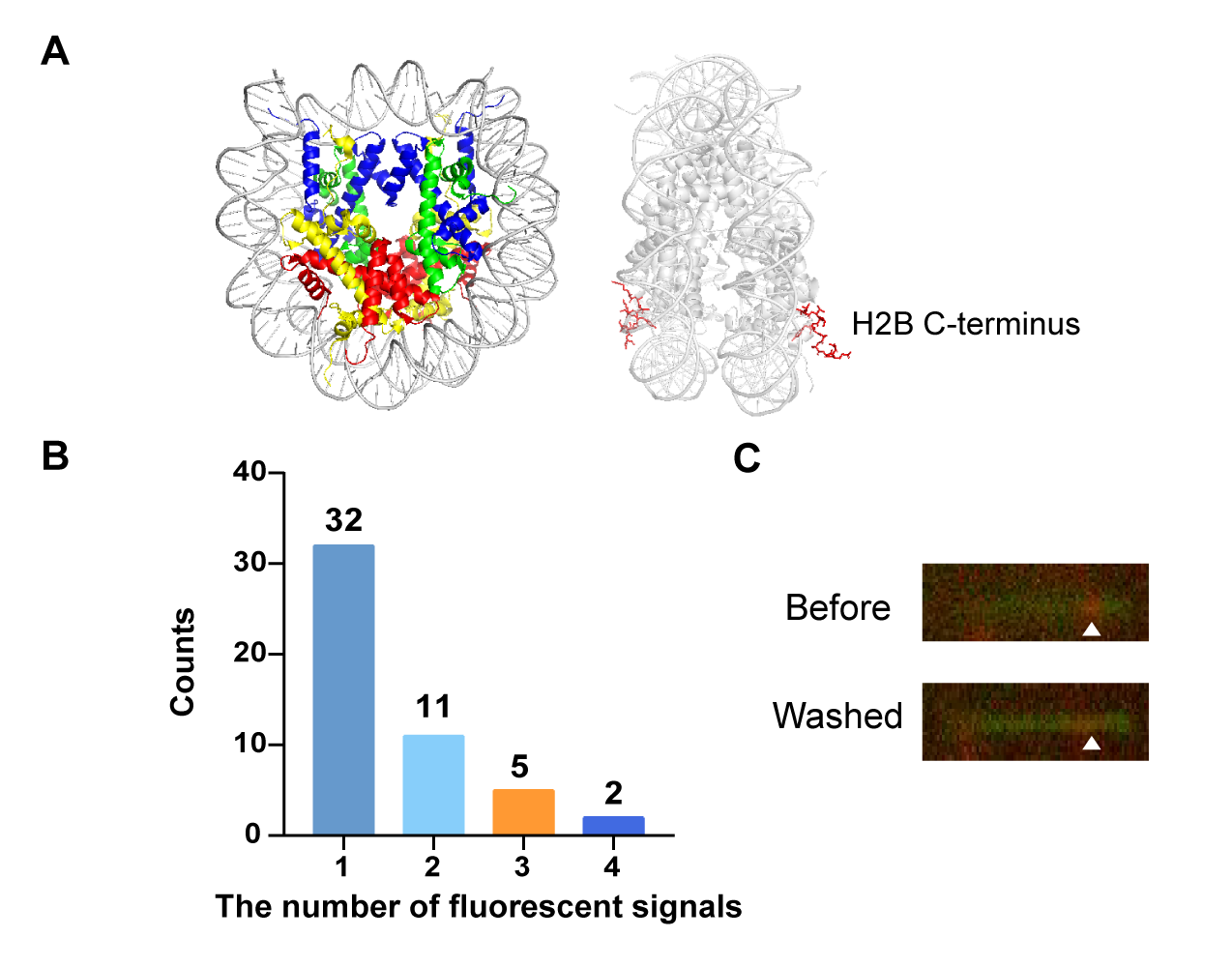

Supplement: Fig. S3 — Fluorescent labeling of nucleosomes. [file mbio.00993-23-s0004.tif]

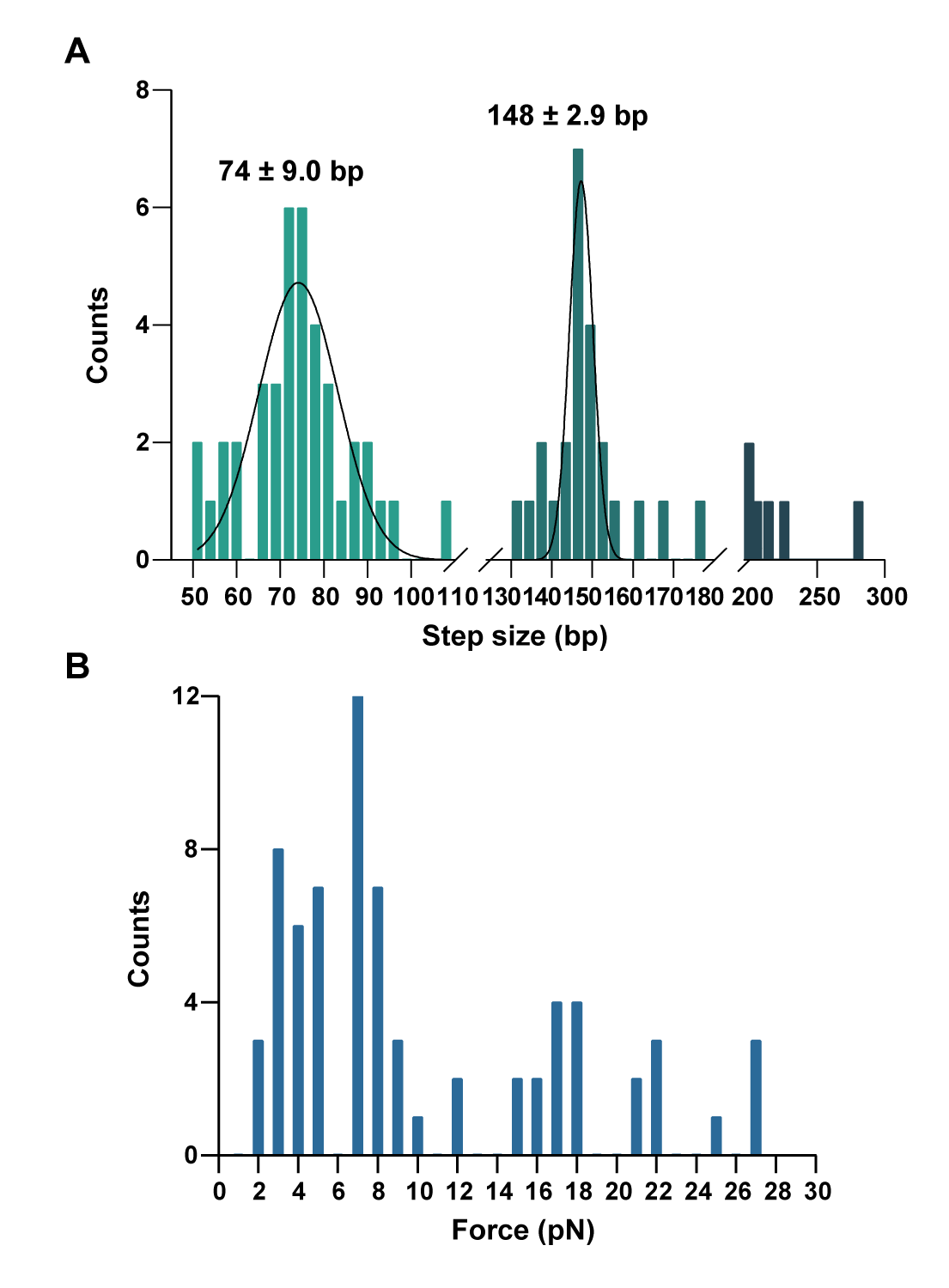

Supplement: Fig. S4 — Histograms of step sizes (bp) and forces (pN) in disruption events during chromatin stretching in YNPEwt. [file mbio.00993-23-s0005.tif]

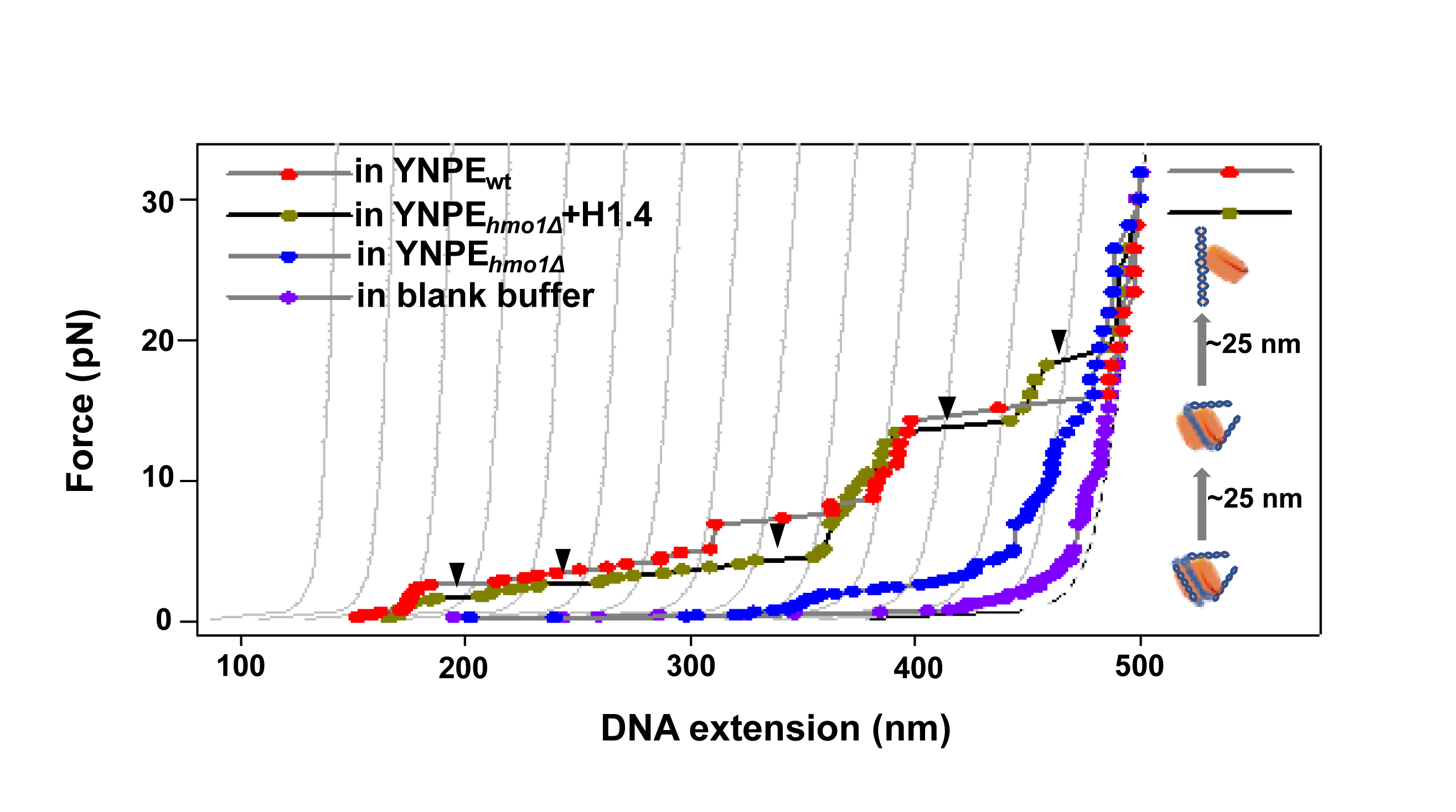

Supplement: Fig. S5 — The representative force-extension curves of chromatin assembled in YNPEhmo1Δ+ H1.4. [file mbio.00993-23-s0006.tif]

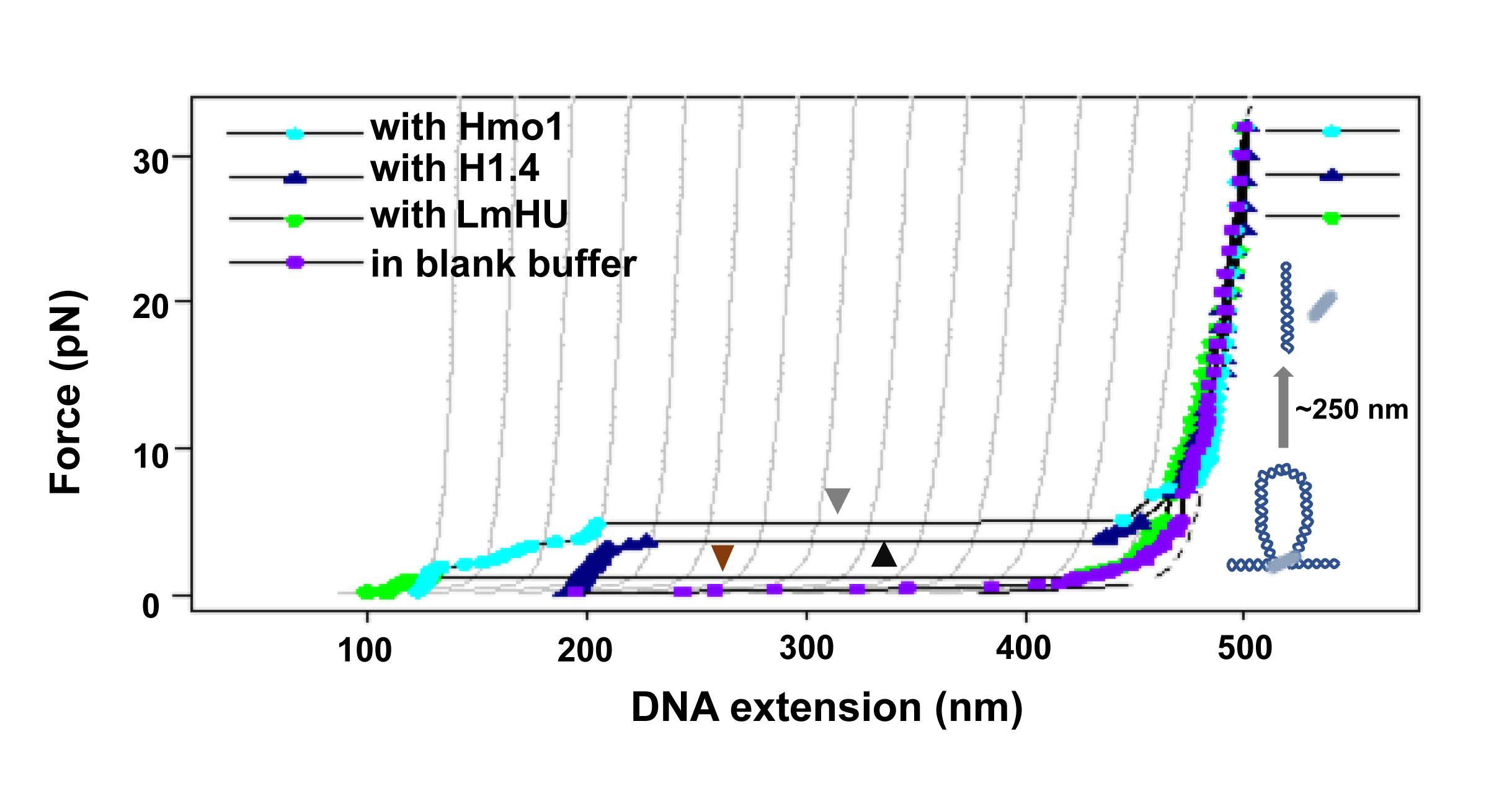

Supplement: Fig. S6 — The representative force-extension curves of dsDNA under different conditions: with purified Hmo1 or H1.4, or LmHU, and in the blank buffer. [file mbio.00993-23-s0007.tif]

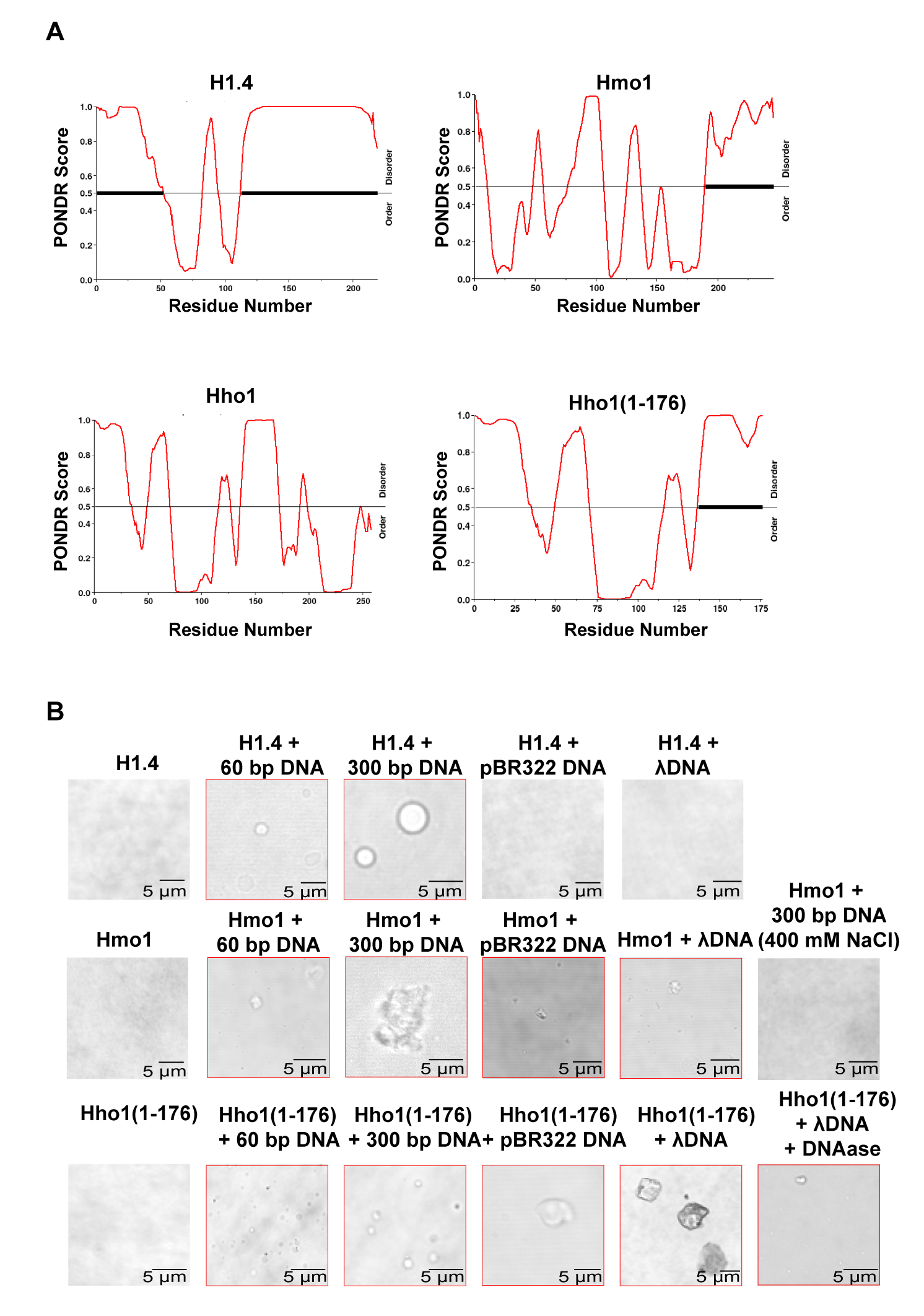

Supplement: Fig. S7 — Hmo1 has a multi-component phase separation property. [file mbio.00993-23-s0008.tif]

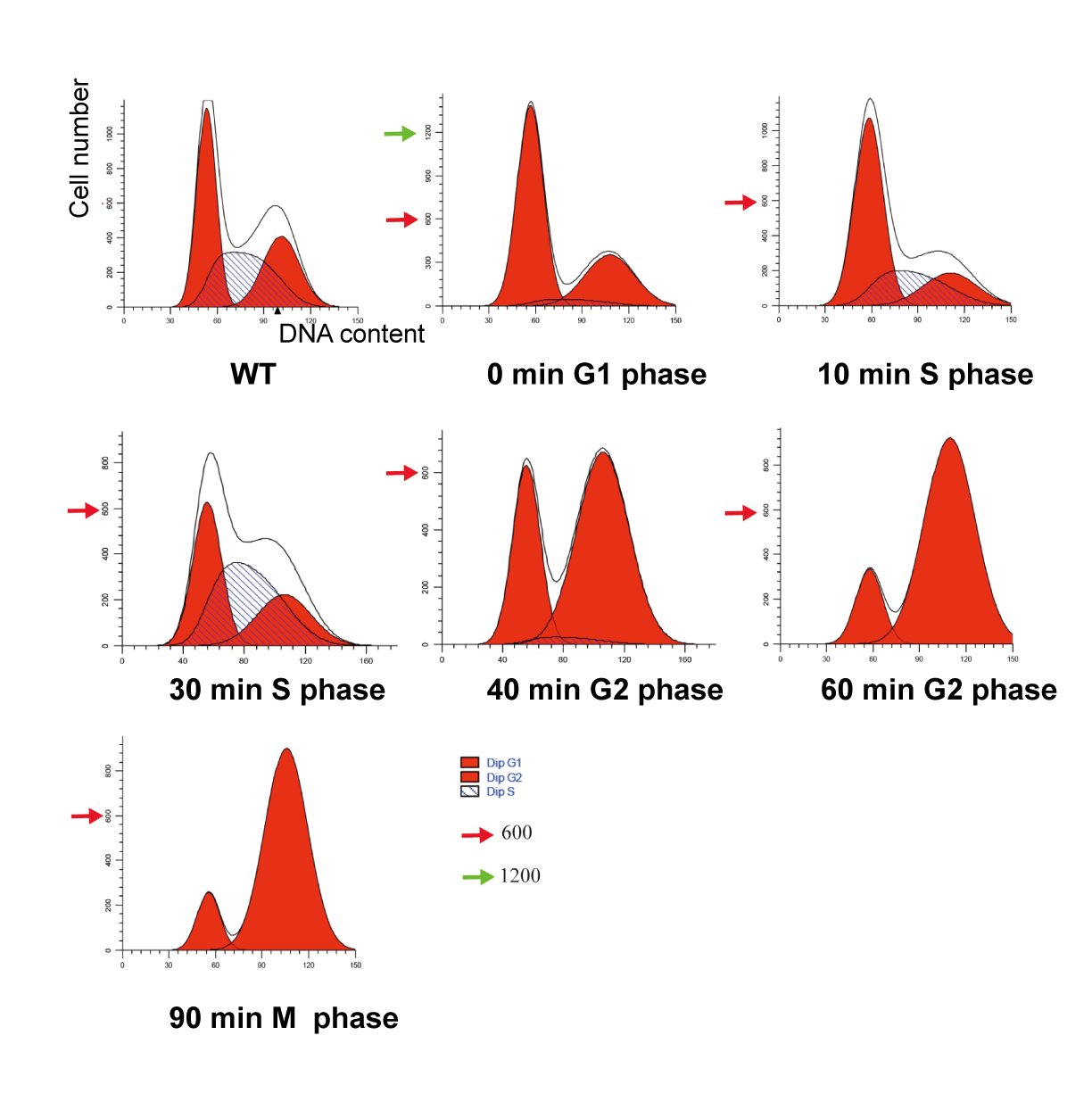

Supplement: Fig. S8 — Analysis of the budding yeast cell cycle by flow cytometry. [file mbio.00993-23-s0009.tif]
